# Earliest Operation for Gall-Stones

**Published:** 1914-08

**Authors:** 


**Earliest Operation for Gall-Stones,** was done by Dr. John S. Bobbs of Indianapolis, June 15, 1867, on Mrs. Mary E. Burnsworth of McCordsville, 40-50 stones, from the size of a shot to that of a pea being removed. Dr. Bobbs died May 1, 1870, aged 61. The patient died in April, 1914, at the Deaconess Hospital of Detroit. Detroit Med. Jour., May, 1914.

